# Supplementary material for: NAT10, an RNA Cytidine Acetyltransferase, Regulates Ferroptosis in Cancer Cells
Source: Antioxidants (Basel). 2023 May 18;12(5):1116. doi: 10.3390/antiox12051116 (PMC10215874; doi:10.3390/antiox12051116)
Supplement: Supplementary file 1 [file antioxidants-12-01116-s001.zip › Supplementary figures.pdf]

Supplementary figures

NAT10, an RNA cytidine acetyltransferase regulates ferroptosis in cancer cells

Mahmood Hassan Dalhat[1,2], *Hani Choudhry*[1,2], *Mohammad Imran Khan*[1,2*]
[1] Department of Biochemistry, King Abdulaziz University, Jeddah 21589, Saudi Arabia
[2] Centre for Artificial Intelligence in Precision Medicines, King Abdulaziz University, Jeddah 21589, Saudi Arabia
*Correspondence: mikhan@kau.edu.sa

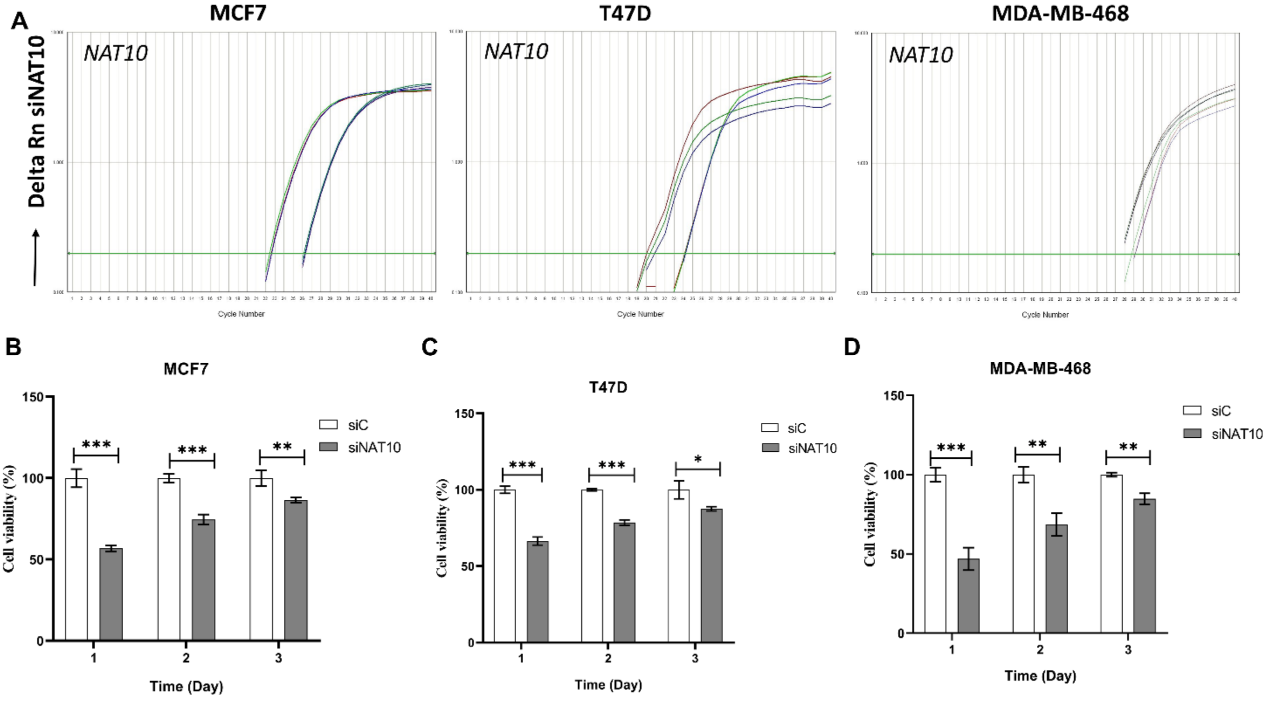

Figure S1. (A) Amplification plot of breast cancer cells transfected with siRNA. Cell viability analysis of NAT10 depleted cancer cells (B) MCF (C) T47D (D) MDA-MB-468. Data are represented as mean±SEM (n=3) and p-value is represented as *p<0.05; **p<0.01; and ***p<0.001.

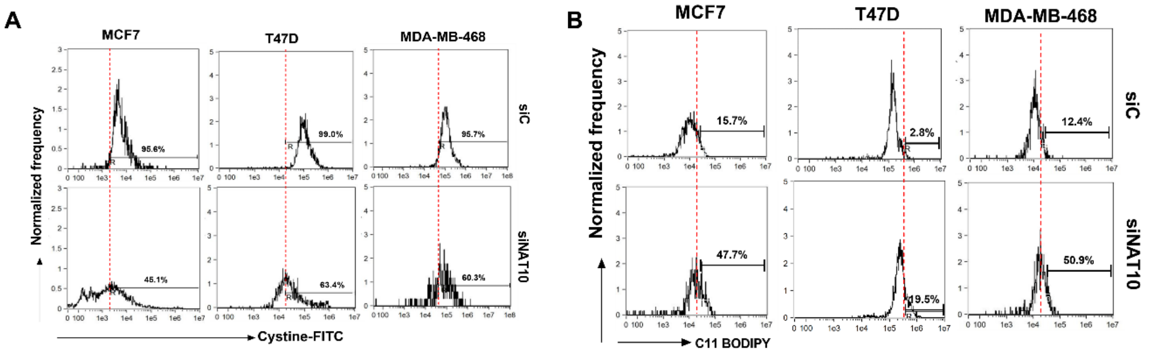

Figure S2. Representative histograms from flow cytometric analysis of (A) Cystine uptake (Cystine-FITC), (B) Lipid peroxidation (C11-BODIPY) in NAT10 depleted cancer cells.

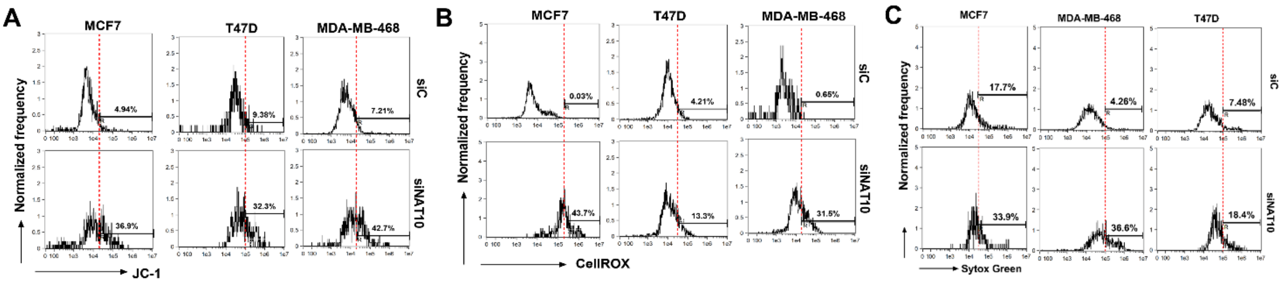

Figure S3. Representative histograms from flow cytometric analysis of (A-C) mitochondrial membrane potential, cell ROS and cell death assay of NAT10 depleted cancer cells.

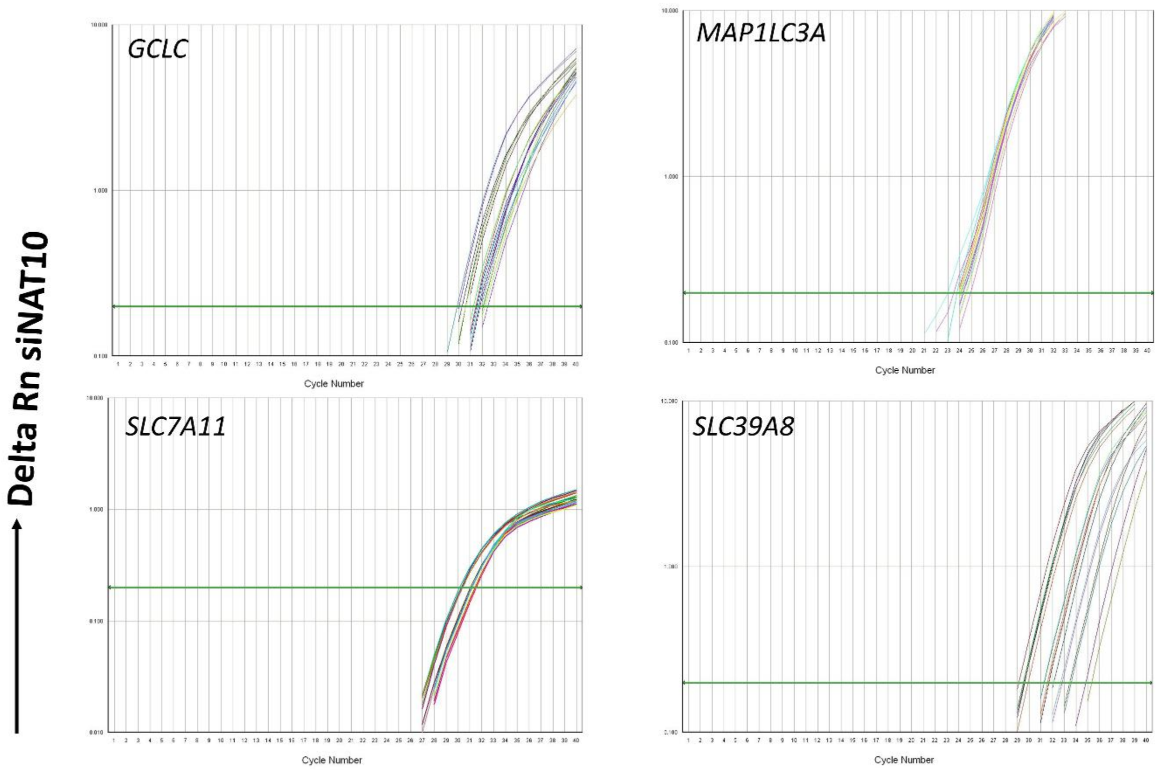

Figure S4. Amplification plot from RIP-PCR of ferroptosis genes in NAT10 depleted cancer cells.

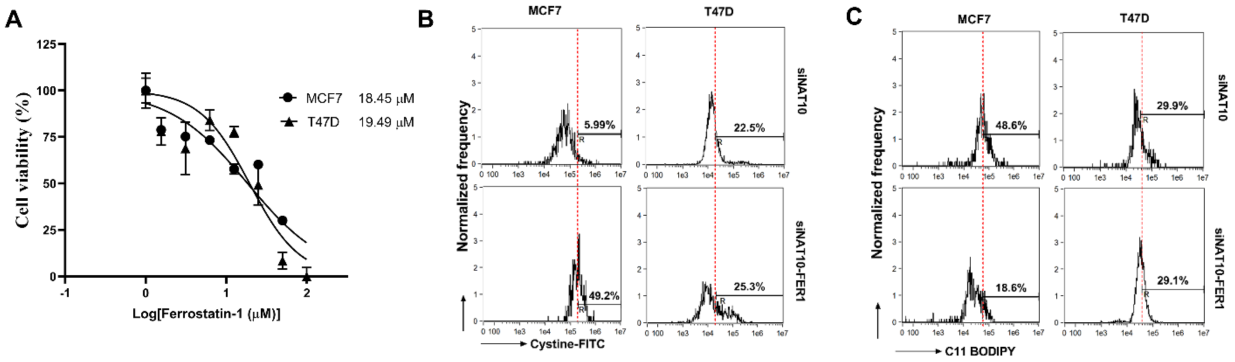

Figure S5. Impact of ferrostatin-1 on NAT10 depleted cancer cells. (A) Cell viability assay of ferrostatin (fer-1) treated cancer cells after 24 h. (B, C) Representative histograms from flow cytometric analysis of cystine uptake and lipid peroxidation of NAT10 depleted cancer cells followed by 24 h treatment with fer-1.

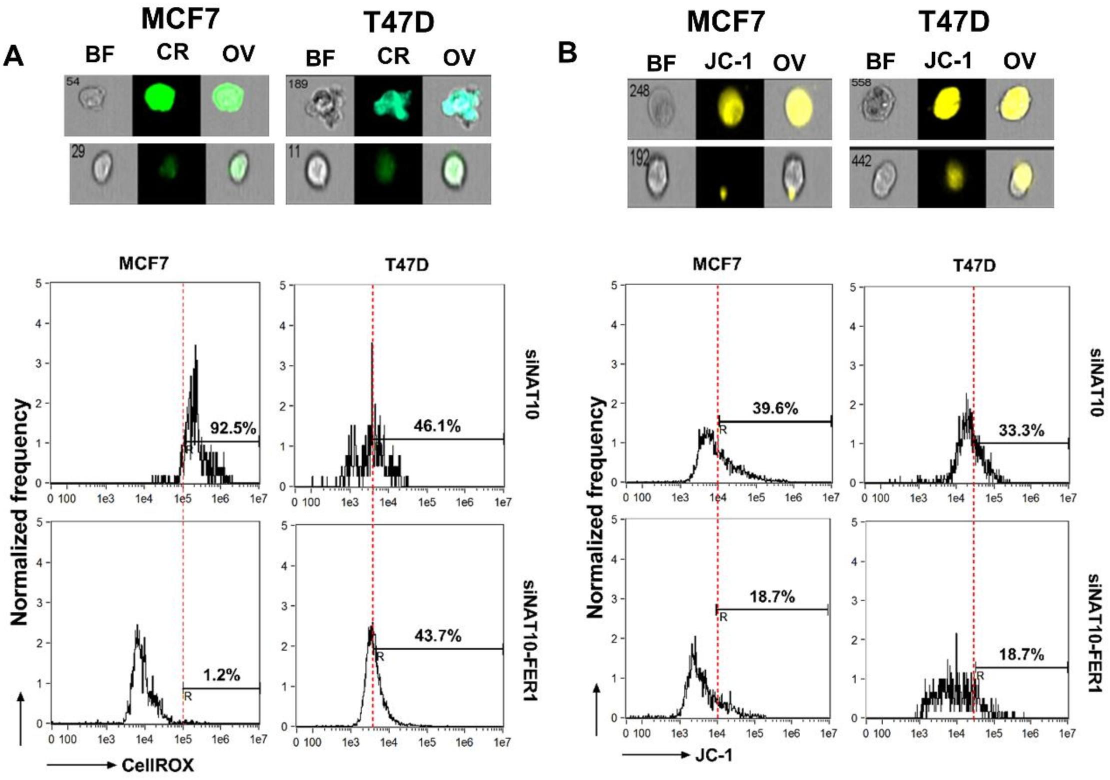

Figure S6. Flow cytometric analysis of (A) Cell ROS and (B) Mitochondrial membrane potential, in NAT10 depleted cancer cells treated with ferrostatin-1 (fer-1). BF: Brightfield, CR: CellROX; JC-1; JC-1 stain of cell with depolarized mitochondria, OV: Overlay.

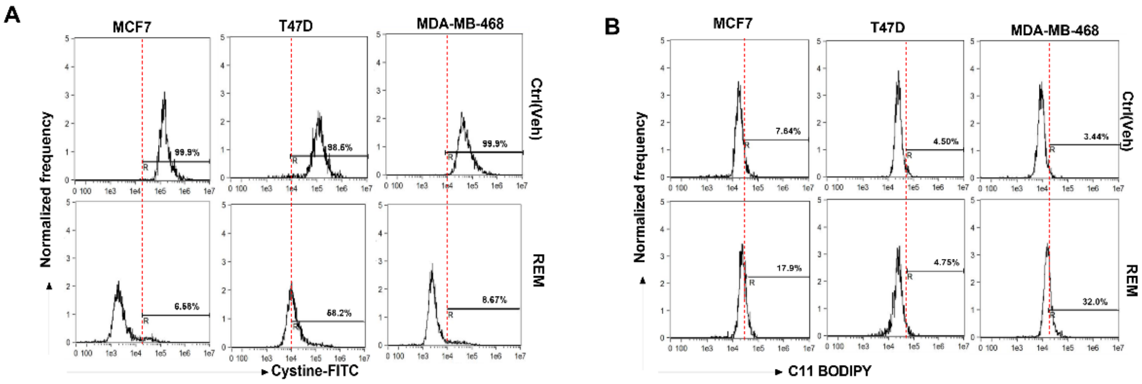

Figure S7. Representative histograms from flow cytometric analysis of (A) Cystine uptake (Cystine-FITC), (B) Lipid peroxidation (C11-BODIPY) in cancer cells 24 h posttreated with Remodelin.

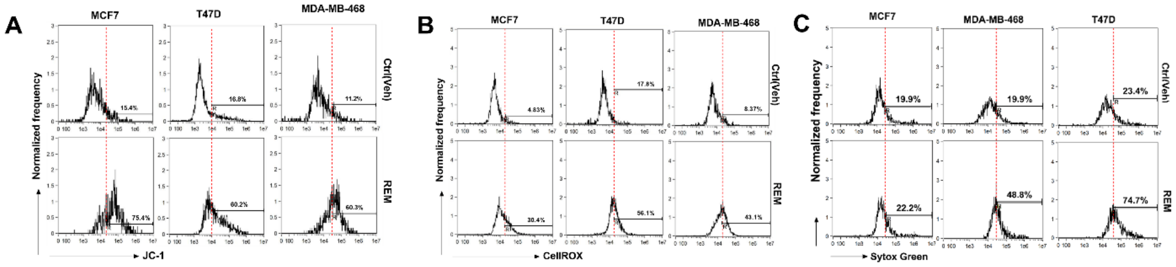

Figure S8. Representative histograms from flow cytometric analysis of (A-C) mitochondrial membrane potential, cell ROS and cell death assay of Remodelin treated cancer cells after 24 h.

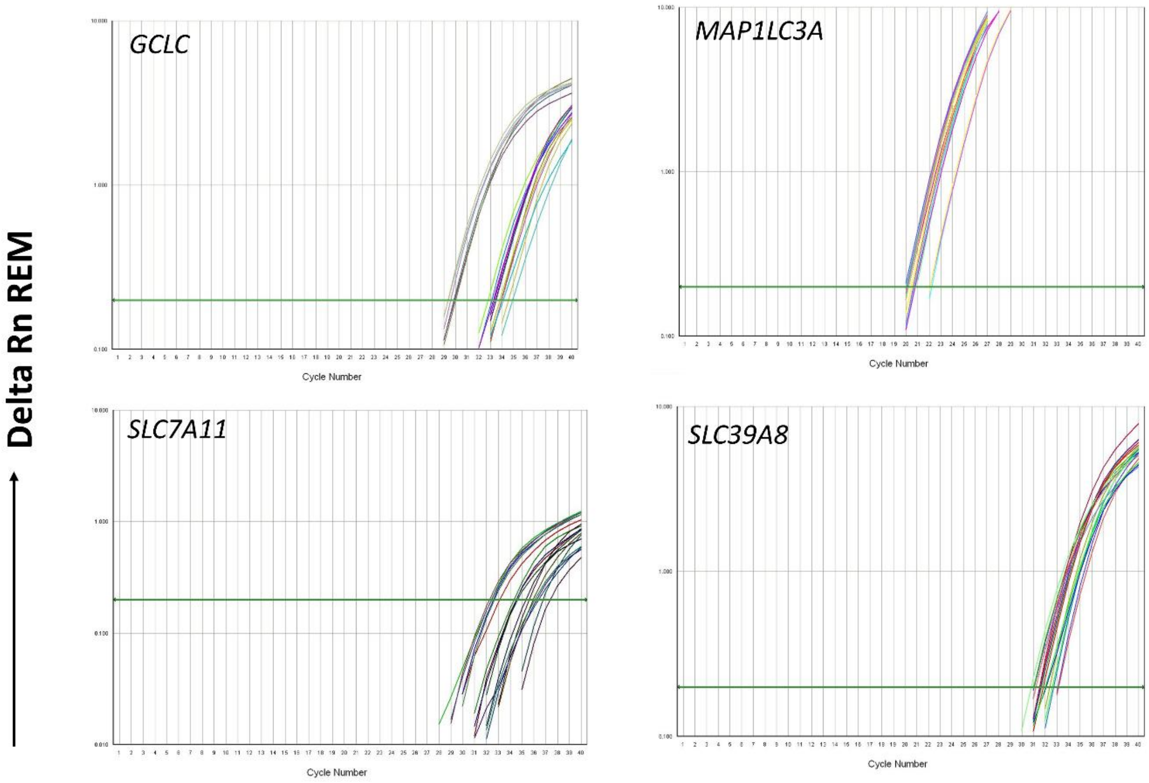

Figure S9. Amplification plot from RIP-PCR of ferroptosis genes in Remodelin treated cancer cells.

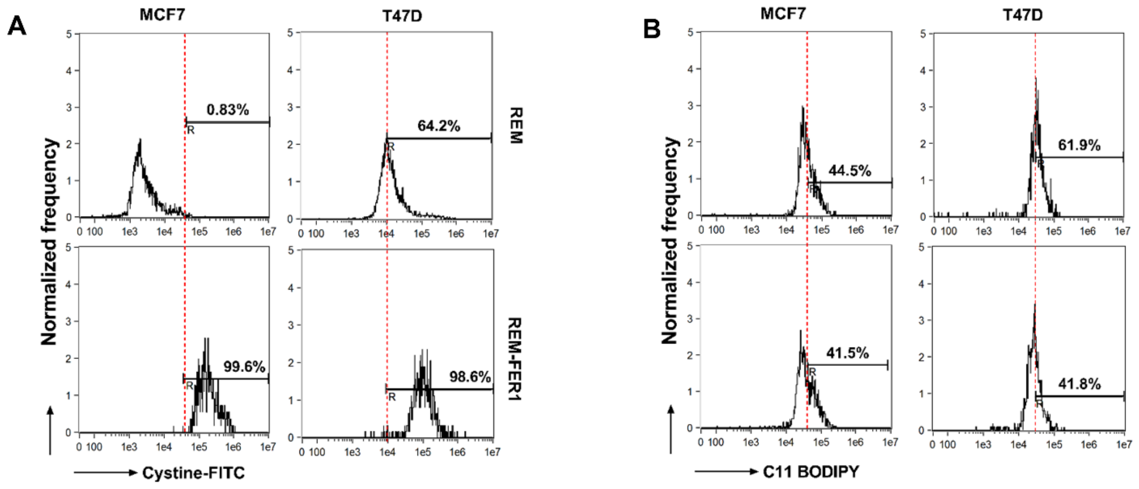

Figure S9. (A, B) Representative histograms from flow cytometric analysis of cystine uptake and lipid peroxidation of 24 h Remodelin treated cancer cells subsequently followed by 24 h treatment with fer-1.

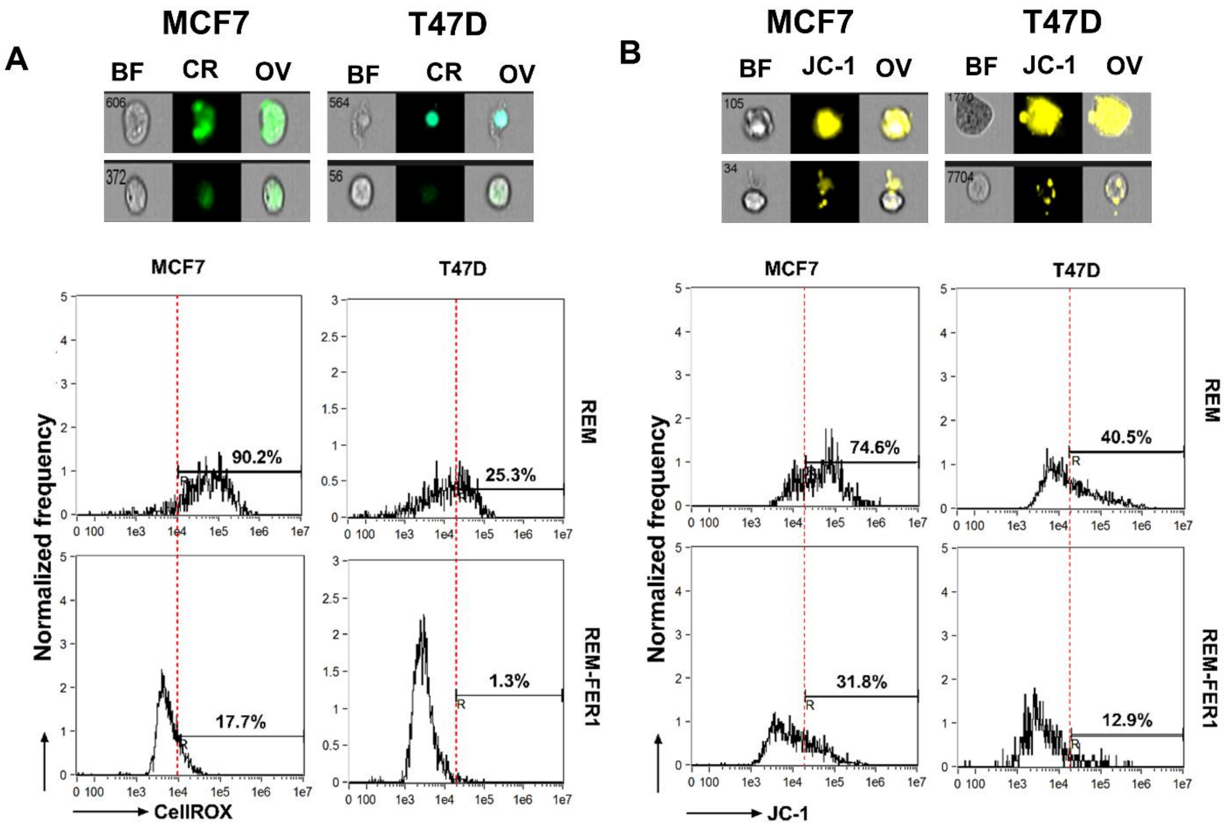

Figure S11. Flow cytometric analysis of (A) Cell ROS and (B) Mitochondrial membrane potential, in Remodelin treated cancer cells treated with fer-1. BF: Brightfield, CR: CellROX; JC-1; JC-1 stain of cell with depolarized mitochondria, OV: Overlay.
