# Supplementary material for: NAT10, an RNA Cytidine Acetyltransferase, Regulates Ferroptosis in Cancer Cells
Source: Antioxidants (Basel). 2023 May 18;12(5):1116. doi: 10.3390/antiox12051116 (PMC10215874; doi:10.3390/antiox12051116)
Supplement: Supplementary file 1 [file antioxidants-12-01116-s001.zip › Table S1.pdf]

Table S1: Joint pathway analysis of metabolomics and transcrptomics of NAT10 depleted MCF7

S/ N	Pathways	Total	Expected	Hits	Raw p	-Log10 pvalue	Holm adjust	FDR	Impact
1	Adherens junction	71	2.0897	10	3.99E-05	4.3986	0.01322	0.011803	0.30667
2	Cell cycle	124	3.6496	13	7.13E-05	4.1468	0.023534	0.011803	0.57273
3	Cellular senescence	165	4.8564	14	0.000365	3.4379	0.12003	0.030615	0.31624
4	Proteoglycans in cancer	211	6.2103	16	0.000504	3.2974	0.16538	0.030615	0.21
5	Hippo signaling pathway	154	4.5326	13	0.000617	3.2099	0.20165	0.030615	0.33333
6	Ubiquitin mediated proteolysis	136	4.0028	12	0.000669	3.1746	0.21808	0.030615	0
7	cGMP-PKG signaling pathway	177	5.2096	14	0.000739	3.1314	0.24017	0.030615	0.29268
8	mTOR signaling pathway	157	4.6209	13	0.00074	3.1308	0.24017	0.030615	0.39286
9	Ferroptosis	71	2.0897	8	0.001096	2.9601	0.3541	0.038565	0.081081
10	Insulin resistance	128	3.7674	11	0.001378	2.8606	0.44385	0.038565	0.27941
11	RNA transport	168	4.9447	13	0.001384	2.8589	0.44419	0.038565	0.23853
12	mRNA surveillance pathway	91	2.6784	9	0.001398	2.8545	0.4474	0.038565	0.23214
13	Human papillomavirus infection	333	9.801	20	0.002017	2.6953	0.64334	0.05135	0.21875
14	FoxO signaling pathway	136	4.0028	11	0.002241	2.6496	0.71263	0.052983	0.21429
15	Focal adhesion	201	5.9159	14	0.002486	2.6045	0.78811	0.053213	0.62903
16	PPAR signaling pathway	81	2.384	8	0.002572	2.5897	0.81282	0.053213	0.27119
17	Herpes simplex virus 1 infection	494	14.54	26	0.002975	2.5265	0.93716	0.057754	0.090909
18	Hepatitis B	163	4.7975	12	0.003162	2.5	0.99295	0.057754	0.15217
19	Dopaminergic synapse	143	4.2088	11	0.003315	2.4795	1	0.057754	0.31944
20	Endocytosis	255	7.5053	16	0.003613	2.4422	1	0.059788	0.051095
